# Survival Rates of Anterior-Region Resin-Bonded Fixed Dental Prostheses: An Integrative Review

**DOI:** 10.1055/s-0041-1731587

**Published:** 2021-08-24

**Authors:** José Manuel Mendes, Anne Le Guen Bentata, Juliana de Sá, António Sérgio Silva

**Affiliations:** 1 Dental Science Department, Institute for Research and Training Advanced in Health Sciences and Technologies, Gandra, Portugal; 2 Department of Oral Rehabilitation, University Institute of Health Sciences Rua Central da Gandra, Gandra, Portugal

**Keywords:** bridge, cantilever, fixed dental prostheses, resin-bonded, survival rate

## Abstract

This study aimed to review clinical publications involving anterior-region resin-bonded fixed partial dentures to evaluate their survival rates vis-à-vis their materials and design. An electronic search was conducted using PubMed/MEDLINE to identify articles that reported on the longevity of anterior resin-bonded fixed dental prostheses published between 2000 and 2020. Only primary clinical studies that involved a follow-up after at least 3 years were included in this review. A statistical analysis was performed to evaluate resin-bonded fixed dental prostheses’ survival rates in relation to their materials and design. This review ultimately included 23 clinical publications, comprising prospective studies, retrospective studies, and randomized controlled trials. Its statistical analysis estimated the studied prostheses’ 5-year survival rate at 86.2% for metal-framed prostheses, 87.9% for zirconia prostheses, 93.3% for alumina prostheses, 100% for glass or ceramic prostheses, and 81.7% for fiber-reinforced composite restorations. Failure rates did not significantly differ between the different material groups or between the single- and double-retainer groups. Resin-bonded fixed dental prostheses present excellent 5-year clinical longevity in the anterior sector and a favorable benefit/risk/cost ratio. Currently, no consensus has been established on an ideal material for these restorations. Cantilever design tends to limit constraints on the prostheses’ retainers and, thus, increases their survival time. All-ceramic cantilever fixed partial dentures can be considered as a definitive therapy, given their high success and survival rates. They are an optimal solution for adolescents or young adults facing potentially continuous growth.

## Introduction

The congenital absence of teeth is among the most common developmental disorders. 1 Tooth agenesis has been estimated to affect 8% of a Portuguese population studied at Porto’s Faculty of Dentistry. The most frequently missing teeth in this study, excluding the third molars, were the mandibular second premolars (28.6%) and the maxillary lateral incisors (27.8%). 2 Moreover, the traumatic absence of teeth is also highly frequent, especially among children and young adults. An observational study of a randomized sample of 301 students, aged between 15 and 19 years, who were attending public secondary schools in Porto reported a 44.2% prevalence of dental trauma. The most affected teeth in this study were the maxillary central incisors, especially among male participants. 3 Thus, dentists commonly encounter missing teeth in the anterior aesthetic region and must be proficient in various treatment strategies, depending on their patients’ characteristics (age, medical conditions, and economic resources).

Several therapeutic options are available to treat unitary anterior edentulism, including orthodontic space closure, followed by dental recontouring, implant-supported single crowns, conventional fixed partial dentures, adhesive dentures, and removable partial dentures. Resin-bonded fixed partial dentures have traditionally been included among the therapeutic options of this condition since the 1970s. In 1973, Rochette described a two-retainer prosthesis with a metal framework. Later, the University of Maryland improved resin-bonded fixed dental prostheses’ (RBFDPs’) retention through the micromechanical retention of electrolytically etched metal wings. A significant meta-analysis conducted by Pjetursson in 2008 estimated an 87.7% 5-year survival rate for RBFDPs with metal frames. 4 In the early 1990s, Kern et al described the first all-ceramic RBFPD particularly designed to overcome the aesthetic problems associated with metal prostheses in the anterior sector. After various tests on the ceramic type, retainer designs and amounts, and abutment teeth preparation, Kern et al stated in 2017 that “all-ceramic cantilever RBFDPs provide an excellent minimally invasive treatment alternative to implants and conventional prosthetic methods when single missing anterior teeth need to be replaced” and involve a 10-year survival rate of 98.2%. 5

The current study aimed primarily to review the literature on anterior-region RBFDPs’ survival rates to consolidate clinical evidence of the influence of these prostheses’ materials and designs on their survival. Accordingly, the null hypotheses tested were that the studied RBFDPs’ designs or materials would not affect their longevity.

The study’s secondary objectives were to verify whether the survival rates of anterior RBFDPs were comparable to the corresponding rates of unitary implants and whether this therapy can be considered as a definitive solution or only a temporary solution. (Five-year survival rates have been estimated at 98.3% for metal-ceramic implant-supported single crowns and at 97.6% for zirconia implant-supported single crowns. 6 )

## Materials and Methods

### Search Strategy

An electronic search was conducted using PubMed/MEDLINE to identify publications that reported on anterior resin-bonded fixed partial denture survival rates between 2000 and 2020. The following combination of keywords was used: “resin bonded” *or* “ceramic bonded” *and* “bridge” *or* “cantilever” *or* “fixed dental prostheses” *or* “fixed partial denture” *or* “RBBs” *or* “RBFDPs.”

Two operators independently selected the resulting pertinent articles based on their titles and abstracts. This selection also relied on the following criteria for inclusion: primary clinical studies with a minimum 3-year follow-up (prospective or retrospective studies and randomized clinical trials), English as a publication language, the involvement of human subjects, and the availability of abstracts. Moreover, the “related articles” suggested by PubMed, as well as selected reviews’ bibliographies, were also used to identify additional relevant articles. Ultimately, a list of 23 articles was developed from which to extract data about anterior RBFDPs’ survival rates for this study.

### Statistical Analysis

Statistically, RBFDPs’ *success rates* correspond to the percentage of protheses still *in situ* after a certain number of years—without any complication that required a dentist’s intervention. *Survival rates* in this research context are defined as the percentage of restorations still in place after a certain number of years—with or without a practitioner’s intervention and treating any condition (such as a fracture or mobility). Definitions of *success* and *survival rates* may vary from study to study. Therefore, in this review, to standardize longevity calculations, we defined RBFDPs’ *success* as their presence in patients’ mouths, in good functional and aesthetic condition, without any necessary intervention during the revised studies’ follow-up times. Events such as debonding and ceramic chipping of the pontic (even minor occurrences) were considered triggers for RBFDPs’ failure. For example, cases of debonding—even if successful rebonding subsequently occurred—and of ceramic chipping-off resolved by polishing were considered as modifications during the reviewed studies’ observation times and, consequently, registered as failures. We selected this approach to recording complications to more accurately compare studies despite its unfavorable impact on our final quantitative result for RBFDPs’ longevity.

To compare the clinical survival of our reviews’ various cohorts despite their varying number of patients and follow-up times, we calculated RBFDPs’ success rates from the basic data extracted from the reviewed studies. Each reviewed study’s total exposure time was calculated by multiplying its number of RBFDPs involved by its mean observation time. A failure rate per year was then estimated as a percentage, based on the quotient of the number of failures observed over a reviewed study’s total exposure time. Finally, 5-year success rates—or 3-year success rates, in the cases of reviewed studies with shorter effective follow-up times—were respectively obtained using the following formula: 100–5*(failure rate per year) and 100–3*(failure rate per year). These results were then statistically analyzed to estimate 5-year success rates by RBFDP materials and designs. Two analysis of variance tests were run to check for any statistically significant difference between groups.

## Results

### Study Selection

**Fig. 1:**
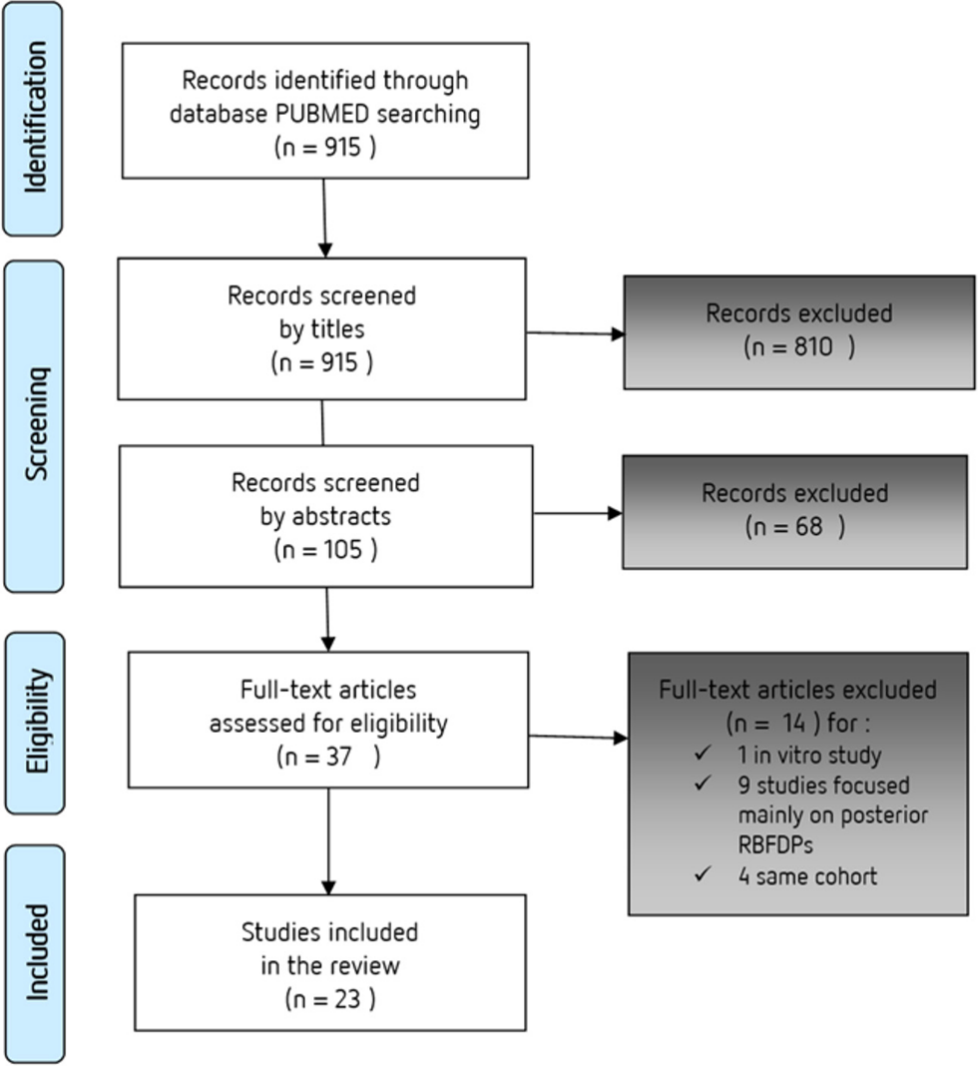
Preferred Reporting Items for Systematic Reviews and Meta-Analyses (PRISMA) flow diagram for search strategy. RBFDPs, resin-bonded fixed dental prostheses.

Our initial electronic search yielded 915 results, which were all screened manually by title. Of these initial results, 810 were rejected and 105 were reviewed, based on their abstracts. Next, 37 studies were assessed as full-text articles, of which 23 studies were included in this review and 14 studies were excluded for the following reasons: one study was conducted *in vitro* , nine studies focused mainly on posterior RBFDPs (premolars and molars), and four studies involved the same cohorts as two follow-up studies that have already been included in our selection. 5 7 The flowchart presented in Fig. 1 outlines this selection process.

### Study Characteristics

This systematic review included 23 studies ( Table 1 ), comprising 10 prospective studies, 7 8 9 10 11 12 13 14 15 16 11 retrospective studies, 5 17 18 19 20 21 22 23 24 25 26 one mix of a prospective trial and a retrospective evaluation, 27 and one randomized controlled trial. 28 In total, we evaluated 2,377 patients with 1,746 anterior fixed partial dentures. From the 23 studies that met this review’s inclusion criteria, the following data were extracted ( Table 2 ):

**Table 1:** Main characteristics of the 23 reviewed studies

Year	First author	Type of study	Total no of patients	Mean age of patients	Drop-out %	Total no of anterior RBFDPs
2020	Naenni et al 20	Retrospective	15	32.4	33	10
2018	Shahdad et al 8	Prospective	26	NR	0	37
2017	Kern 7	Retrospective	87	32	7	100
2016	Kern	Prospective	16	33.3	0	22
2016	Botelho et al 9	Prospective	28	50.5	21	23
2016	Klink and Hüttig 10	Prospective	18	33	0	23
2016	Tanoue 11	Prospective	226	NR	NR	85
2015	King et al 17	Retrospective	805	NR	23	552
2015	Kumbuloglu and Özcan 12	Prospective	134	42	0	175
2014	Botelho et al 19	Retrospective	153	55.4	NR	111
2014	Saker et al 28	Randomized	40	36.1	0	40
2014	Galiatsatos and Bergou 13	Prospective	49	NR	0	54
2013	Lam et al 18	Retrospective	78	NR	0	32
2013	Spinas et al 14	Prospective	30	15	0	32
2013	Younes et al 22	Retrospective	37	32.2	32	24
2013	Sailer et al 21	Retrospective	40	NR	30	20
2013	Sun et al 15	Prospective	35	42.1	0	35
2012	Boening and Ullmann 23	Retrospective	44	22	21	56
2009	van Heumen et al 27	Mix Prospective trial/retrospective evaluation	52	35	27	46
2008	Aggstaller et al 16	Prospective	184	NR	64	84
2006	Garnett et al 24	Retrospective	45	17.6	43	73
2005	Chai et al 25	Retrospective	168	NR	36	33
2000	Corrente et al 26	Retrospective	67	42.1	NR	61
	Total	–	2,377	–	–	1,746
Abbreviations: RBFDPs, resin-bonded fixed dental prostheses; NR, not reported.						

**Table 2:** RBFDP material and design—bonding material

Year	First author	RBFDP material	RBFDP design	Bonding material
2020	Naenni et al 20	Zirconia (Cadcam)	One retainer	Panavia 21 TC
2018	Shahdadet al 8	Zirconia (Cadcam)	One retainer	Multilink Automix
2017	Kern 7	Zirconia (Cadcam)	One retainer	Panavia 21 TC Multilink Automix Zirconia Primer
2016	Kern 5	In Ceram alumina (14) In Ceram zirconia (8)	One retainer	Panavia 21 TC
2016	Botelho et al 9	METAL veneered with ceramic	One retainer (13) Two retainers (10)	Panavia Ex Panavia 21
2016	Klink and Hüttig 10	Zirconia	One retainer	Multilink (22) Variolink (2)
2016	Tanoue 11	METAL veneered with ceramic	Two retainers > Two retainers	Superbond Panavia
2015	King et al 17	METAL veneered with ceramic	Different designs	Panavia 21 TC
2015	Kumbuloglu and Özcan 12	Fiber reinforced composite	Two retainers	Variolink Multilink Rely X Bifix DC
2014	Botelho et al 19	METAL veneered with ceramic	One retainer	Panavia ex Panavia 21
2014	Saker et al 28	METAL Cr-Co alloy (20) IN Ceram alumina (20)	One retainer	Panavia 21 TC
2014	Galiatsatos and Bergou 13	IN Ceram alumina	Two retainers	Variolink II
2013	Lam et al 18	METAL veneered with ceramic	One retainer	Adhesive resin cement
2013	Spinas et al 14	Fiber reinforced composite	Two retainers	Permamix
2013	Younes et al 22	METAL veneered with ceramic	Two retainers	Panavia Ex Panavia 21
2013	Sailer et al 21	Glass ceramic emax	One retainer	Tetric Ceram Rely X Panavia F HFO Variolink
2013	Sun et al 15	Glass ceramic emax	One retainer	Variolink
2012	Boening and Ullmann 23	METAL veneered with ceramic	Two retainers > Two retainers	Panavia ex Panavia 21
2009	van Heumen et al 27	Glass fiber reinforced composite	Two retainers	Compolute Variolink Twinlook Panavia
2008	Aggstaller et al 16	METAL veneered with ceramic	Different designs	Microfill Pontic
2006	Garnett et al 24	METAL veneered with ceramic	One retainer (62) Two retainers (11)	Compolute Variolink Twinlook Panavia
2005	Chai et al 25	METAL veneered with ceramic	One retainer (18) Two retainers (15)	Panavia Panavia Ex Panavia 21
2000	Corrente et al 26	METAL veneered with ceramic/resin	Two retainers	Panavia Ex
Abbreviation: RBFDP, resin-bonded fixed dental prosthesis.

- Total number of anterior-sector RBFDPs (incisors, canines/maxilla, and mandible); this figure accounts for the number of patients with RBFDPs who withdrew from their cohort studies during the follow-up periods (cf. the drop-out percentage in Table 1 ); for articles that referred to both anterior and posterior prostheses, 11 18 21 22 only RBFDPs located in the incisor/canine sector were considered
- Mean exposure time (in years)
- Number of and reason for failures; the following two event categories were defined as RBFDP *failures* :
  - Technical complications, including debonding, pontic fractures, retainer fractures, pontic chipping, and aesthetic complaints
  - Biological complications, including caries, periodontal problems, and tooth movement
- Prosthesis material
- Design (number of retainers)
- Abutment teeth preparation
- Bonding material

### Individual Studies’ Results

The reviewed studies’ 5-year estimated success rates—or 3-year success rates, in the cases of reviewed studies with shorter effective follow-up times—were calculated individually, according to the statistical method described previously in 2.2 ( Table 3 ).

**Table 3:** Estimated success % after 5 years (a% after 3 years)

Year	Author	Total no of anterior RBFDPs	Mean follow-up time (years)	No of failures	Total RBFPD exposure time	Estimated failure rate (%/year)	Estimated success after 5 years (%)
2020	Naenni et al 20	10	11	2	110	1.82	94.55
2018	Shahdad et al 8	37	3	8	111.0	7.21	78.38 ^a^
2017	Kern 7	100	07.7	6	768.3	0.78	96.10
2016	Kern	22	15.6	2	343.2	0.58	97.09
2016	Botelho et al 9	23	18	9	414	2.17	89.13
2016	Klink and Hüttig 10	23	3	4	69	5.80	82.61 ^a^
2016	Tanoue 11	85	13.9	NR	NR	NR	90.28
2015	King et al 17	552	13	92	7176	1.28	93.59
2015	Kumbuloglu and Özcan 12	175	5	13	875	1.49	92.57
2014	Botelho et al 19	111	9.4	10	1043.4	0.96	95.21
2014	Saker et al 28	40	2.8	5	113.3	4.41	86.76 ^a^
2014	Galiatsatos and Bergou 13	54	8	9	432	2.08	89.58
2014	Sailer 21	15	4.4	2	66.6	3.00	90.99 ^a^
2013	Lam et al 18	32	9.6	7	307.2	2.28	88.61
2013	Spinas et al 14	32	5	2	160	1.25	93.75
2013	Younes et al 22	24	16	10	NR	1.49	92.56
2013	Sailer et al 21	20	6	0	120	0.00	100.00
2013	Sun et al 15	35	3.9	0	135.8	0.00	100.00 ^a^
2012	Boening and Ullmann 23	56	6.3	8	352.8	2.27	88.66
2009	van Heumen et al 27	46	5	30	230	13.04	34.78
2008	Aggstaller et al 16	84	6.3	11	529.2	2.08	89.61
2006	Garnett et al 24	73	4.9	32	357.7	8.95	55.27
2005	Chai et al 25	33	5.0	6	165	3.64	81.82
2000	Corrente et al 26	61	6.7	13	408.7	3.18	84.10
	Total	1,746		269			
Abbreviations: RBFDPs, resin-bonded fixed dental prostheses; NR, not reported.							

### Results Synthesis

In total, 1,746 anterior RBFDPs were studied in this review. Of this total, 1,152 (66%) had metal frames and 594 (34%) had nonmetal frames (ceramic or fiber-reinforced composites). The reviewed studies included various design configurations. We categorized design types based on their number of retainers: one retainer (i.e., cantilever design), two retainers, and more than two retainers. For 20 studies assessing 1,022 resin-bonded anterior FDPs, we were able to assess the exact number of designs used in the incisor/canine sector; 523 used cantilever fixed dental prostheses (51.2%), 495 used two retainers (48.4%), and 4 used more than two retainers (0.4%).

### Survival Rates by Material and Design

After we performed the statistical method presented in 2.2, we estimated 5-year success rates as follows ( Table 4 ): 86.2% (standard deviation [SD] = 10.9, standard error [SE] = 3.3) for metal-frame RBFPDs, 87.9% (SD = 9.2, SE = 5.3) for zirconia RBFPDs, 93.3% (SD = 5.3, SE = 3.7) for alumina RBFPDs, 100% for glass-ceramic RBFPDs, and 81.7% (SD = 19.9, SE = 11.5) for fiber-reinforced composite RBFPDs. The studied RBFDPs’ frame materials did not have a statistically significant effect on the RBFDPs’ longevity ( *p* = 0.46).

**Table 4:** Estimated success rate by RBFDP material and design

Five-year success rate			
*By framework material*		*By number of retainers*	
Metal	86.2%	One retainer	95.4%
Zirconia	87.9%	Two retainers	85.2%
Alumina	93.3%		
Glass-ceramics	100%		
FR composite	81.7%		
Abbreviations: FR, fiber-reinforced; RBFDP, resin-bonded fixed dental prosthesis.			

Based on all the relevant studies in this review, the cantilever design showed better 5-year longevity than the two-wing design, at 91.9% (SD = 7.4, SE = 2.3) versus 85.2% (SD = 13.4, SE = 5.5), respectively. However, failure rates were not statistically significant among either group ( *p* = 0.22).

This review included several studies, based on a comparison of designs. In two reviewed comparative studies, RBFDPs with metal frames demonstrated significantly better success and survival when designed with a single retainer, rather than two retainers. 9 17 Cantilever fixed partial dentures also showed better results regarding biological complications; for example, “no abutment tooth was lost or endodontically involved.” 9 Single-retainer prostheses’ performance was attributed to their avoidance of differential movement among the abutment teeth, 17 as evidenced in two-winged restorations. All-ceramic RBFDPs’ longevity was largely affected by the restorations’ design. However, two of the reviewed studies did not observe any statistically significant difference in success between designs. 11 25

One reviewed study compared traditional metal-ceramic (cobalt-chromium-ceramic) and all-ceramic (glass-infiltrated alumina In-Ceram) frame material RBFDPs, concluding that survival rate differences between cantilevered metal-ceramic FPDs and all-ceramic FPDs were not significant. 28 Several reviewed studies used zirconia (IPS e.max ZirCad veneered with IPS e.max Ceram), and one study tested other zirconia materials. Some studies selected other types of all-ceramic materials, such as glass-infiltrated alumina 7 13 and lithium disilicate ceramics e.max. 15 21 The mean survival rates for each type of material are summarized in Table 4 . The reviewed frame materials demonstrated no statistically significant effects.

All the reviewed studies agreed in concluding that RBFDPs—and especially cantilevered all-ceramic fixed partial dentures—offer promising clinical survival and functional longevity in the anterior upper and lower sectors. Survival rates—defined as the prostheses’ presence in situ after the reviewed studies’ follow-up periods, with or without intervention—were high in most of the studies. These survival rates are summarized in Table 5 . However, three studies yielded contrasting results with significantly lower survival rates—specifically, the studies by van Heumen et al (two retainers, fiber-reinforced resin composite), 27 Garnett et al (multiple designs, metal cast), 24 and Tanoue (multiple designs, metal cast). 11

**Table 5:** RBFDPs’ survival rates

Study	Design, material of the prosthesis	Follow-up time (y)	Survival rate (%)
Kern 7	Cantilever, zirconia	10	98.2
Kern 2016 5	Cantilever, alumina	18	81.8
Botelho et al 2014 19	Cantilever, metal cast	9.4	90
King et al 17	Multiple designs, metal cast	10	80.4
Galiatsos and Bergou 13	2 retainers, alumina	8	85.2
Sailer et al 21	Cantilever, glass-ceramics e.max	6	100
Kumbuloglu and Özcan 12	2 retainers, fiber-reinforced composite	5	97.7
Sun et al 15	Cantilever, glass-ceramics e.max	4	100
Naenni et al 20	Cantilever, zirconia	10	100
Saker et al 28	Cantilever, all-ceramic / cantilever, metal-ceramic	3	90/100
Klink and Hüttig 10	Cantilever, zirconia	3	100
Abbreviation: RBFDPs, resin-bonded fixed dental prostheses.			

### Complications

**Fig. 2:**
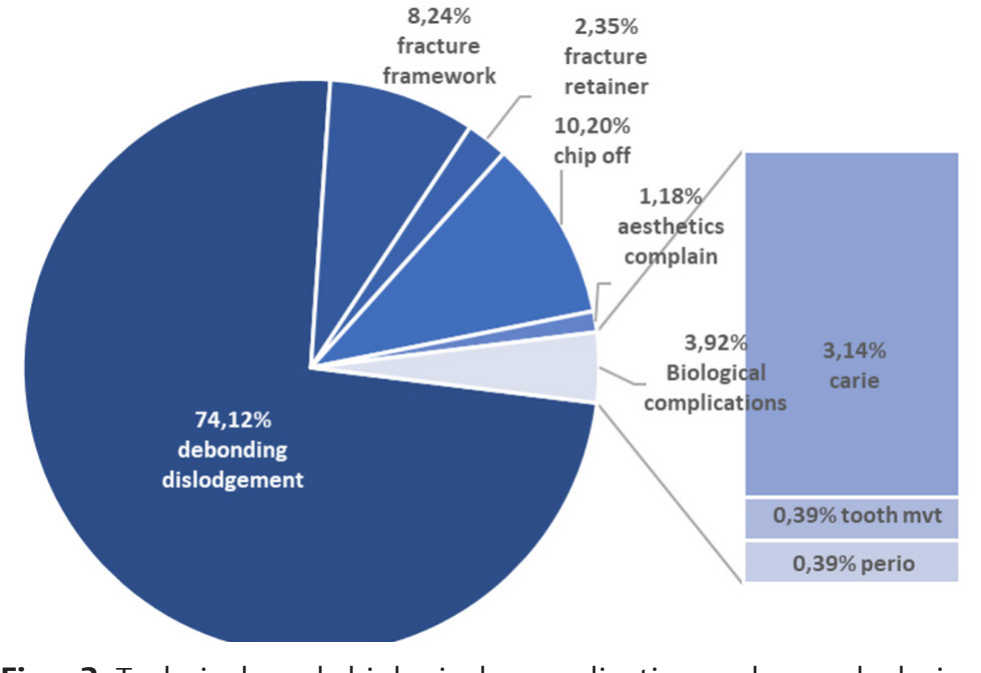
Technical and biological complications observed during follow-up time.

We extracted data on the number of complications encountered during patient follow-up in 22 of the 23 reviewed studies. This analysis reported 279 complications after RBFDP placements in the anterior sector. Moreover, 20 articles reported the nature of these complications. Of the 255 failures specifically identified in this review, 245 (96%) were technical in nature and 10 (4%) were biological in nature. Fig. 2 provides an overview of complications that resulted after RBFDP placement.

Debonding was, by far, the most common reason for resin-bonded fixed partial dentures’ failure. RBFDPs with metal frames seemed to be the most affected by this technical problem. In a long-term prospective study (with an 18-year mean follow-up time), Botelho et al observed that debonding was the only cause of failure among metal-frame RBFDPs used to replace missing maxillary incisors. 9 However, retention rates were highly influenced by the design, as 100% of cantilever fixed partial dentures survived without any complications whereas only 50% of three-unit prostheses survived, only 10% without intervention. 9

Kumbuloglu and Özcan found that fiber-reinforced composite fixed dental prostheses “experienced failures in general were due to debonding of the restoration or delamination of the veneering composite.” 12 However, almost all complications were minor, and after practitioners’ intervention, all but one initial prosthesis remained functioning until the end of the study’s 4.8-year follow-up period. Finally, the authors identified a 97.7% survival rate for composite three-unit RBFDPs.

Evaluating ceramic prostheses, Kern et al reported six debonding incidents (out of seven total failures) for anterior zirconia ceramic RBFDPs. 5 Notably, however, three of these debonding incidents were due to trauma, and all six restorations could be rebonded without further difficulties. The authors claimed that “zirconia ceramic RBFDPs yielded a 10-year survival rate of 98.2%” and, “when debonding was considered a complication, the success rate (survival with complication) was 92.0% after 10 years.” 5 With glass-ceramic cantilever fixed partial dentures, both Sun et al and Sailer et al achieved a 4-year success rate of 100% with no debonding recorded. 15 21

### Abutment Tooth Preparation

RBFDPs are considered a biologically conservative treatment for unitary edentulism. They require minimally invasive preparation and, thus, constitute a reversible treatment. Preparation of the abutment teeth depends on whether RBFDPs are regarded as a provisory measure or a permanent restoration. However, despite this consideration, our literature review highlighted several views of what constitutes appropriate dental preparation before placing a RBFDP. The majority of reviewed studies referred to the creation of grooves, pits, slots, chamfers, and proximal boxes on the lingual/palatal face of the abutment teeth to secure prostheses’ seating and retention. 5 7 9 10 15 19 20 25 28 Although a few of the reviewed authors opted for a “no preparation” option, 8 21 the majority agreed on the benefits of minimal preparation without penetration into the dentine, using a supragingival finish line and allowing an adequate bonding surface for the material chosen for the prostheses. King et al reported a twofold increase in failure when preparation penetrated the enamel. 17 However, the reviewed publications described several surface treatment protocols for prostheses before bonding, including alumina air-abrasion, tribochemical silica-coating, etching with hydrofluoric acid, silanization, ultrasonic cleaning, metal primers, and zirconia primers.

### Patient Outcomes

Patients’ aesthetic satisfaction following rehabilitation with anterior RBFDPs was assessed in four studies included in this review. Botelho et al estimated that “95.2 percent of patients were satisfied with the aesthetics of the prostheses, and patient satisfaction with the overall prosthesis experience was also high.” 9 When comparing two-unit (CL2) and three-unit (FF3) resin-bonded fixed partial dentures, these authors found no significant differences in satisfaction and oral health–related quality of life between the two groups in their study. Nevertheless, the CL2 patients were more favorable about cleaning their prostheses, which allowed for the use of dental floss in the interproximal areas. Similarly, King et al concluded that “the majority of patients rated the function of their restorations as good.” 17 Cases of patients reporting only a “satisfactory” appearance of their restorations were linked to the display of metallic frames’ cervical margins or to the graying effect they could have on the abutment teeth. For all-ceramic RBFDPs, Sun et al evaluated patients’ satisfaction with their restorations’ aesthetic and functional outcomes at their final follow-up after a mean of 46.57 months. 15 These patients were asked to register their satisfaction on a visual analog scale (VAS) from 0 ( *very dissatisfied* ) to 100 ( *very satisfied* ), considering a score above 80 to reflect a high degree of satisfaction. The average VAS score in this study was 87.5, which demonstrates an adequate response from IPS e.max cantilever FPDs to patients’ expectations.

### Dentists’ Experience

Four reviewed studies considered operators’ experiences a significant factor associated with RBFDPs’ success. King et al stated that, “for bridges provided by staff or postgraduate students, the survival rate was just over double that of undergraduate students.” 17 Tanoue also concluded that “the risk of failure […] of inexperienced dentists was 2.0 times greater than that of dentist experienced and specialized in adhesive dentistry.” 11 Botelho et al explained that their statistical analysis showed a longer service life for prostheses placed by full-time staff than prostheses placed by students—though this difference was not significant for either of their study groups regarding debonding rates specifically. 19 Finally, Garnett et al drew similar conclusions, reporting failure risks 3.9 times higher than experienced dentists for junior staff and 2.5 times higher for supervised students. 24

### Various Clinical Factors

The reviewed publications also referred to the following various criteria as relevant or irrelevant for RBFDPs’ clinical success.

- *Patient age at insertion* : Tanoue considered patients’ age at the time of insertion significant, claiming that “the risk of failure in younger patients (age ≤ 56) was 1.7 times greater than that in older patients (age > 56).” 11 This difference was mainly attributed to the young population’s higher risk of trauma. On the contrary, King et al stated that patients under 30 years old demonstrated a lower failure rate than patients over 30 years old (13.7 and 24.2%, respectively). 17
- *Maxilla/mandible location* : The vast majority of reviewed studies reported that RBFDPs’ upper or lower location did not statistically affect their longevity. 8 11 16 17 19 22
- *Bonding system* : The reviewed studies referred to various types of cement, most commonly using PANAVIA EX and PANAVIA 21 by Kuraray. This review does not support a conclusion that one cement is superior to another.
- *Occlusal factors and parafunctional habits* : Klink and Hüttig claimed that “success depends on dynamic occlusal relation.” 10 King et al also reported that the presence of contacts in excursions of the pontic was significantly associated with a higher failure rate. 17 In contrast, the presence of contacts in excursions of the abutment was not significantly associated with RBFDPs’ longevity. 17
- *Rubber dam use* : The importance of moisture control through rubber dam use during RBFDPs insertion was sometimes referred to, but the reviewed studies did not always document the use of a rubber dam. At the Bristol Dental Hospital, King et al reported a significantly higher success rate for RBFDPs placed with a rubber dam. 17 More recently, rubber dams have ceased to be considered an optional clinical factor and, rather, come to be regarded as a mandatory part of the insertion process for restorations.

## Discussion

### A Shift toward All-Ceramic Restorations

This review of dental literature about anterior-zone RBFDPS showed that this type of prosthesis has demonstrated successful clinical results and patient satisfaction. The current trend clearly reflects a shift toward all-ceramic restorations and away from prostheses with metal frames. Recently, more favorable survival rates have been related to RBFDPs’ cantilevered design.

### In Search of an Ideal Material

Since the early 1990s, the dental school of Hong Kong has considered anterior-zone RBFDPS restorations as a standard therapy to offer patients. Botelho and Lam published various long-term studies reporting high survival rates for nickel-chromium RBFDPs, and they also identified reasons for preferring cantilever fixed partial dentures to implant-supported restorations. Lam et al highlighted, in a case series of 78 patients, fewer biological complications resulting from cantilever FPDs (7.7%) than implant-supported crowns (25.6%). 18 However, their conclusion was tempered by the necessity for longer-term follow-up studies, after up to 10 years, to validate RBFDPs’ performance versus unitary implants in the anterior sector.

Moreover, a survey showed that 94.4% of questioned dentists described themselves as “confident” or “very confident” in providing metal cantilever fixed partial dentures. 29 However, from patients’ perspective, metal-based restorations may lead to aesthetic problems due to their metal’s grayish shine, which is particularly annoying when these prostheses are placed in the anterior zone. Moreover, the allergenic, corrosive, and even mutagenic effects of certain nonprecious metals have been discussed. These concerns have led to a search for changing and improved materials for use in resin-bonded prostheses.

In recent years, shifts in modern adhesive dentistry have trended toward the use of ceramics—a highly biocompatible material. The first attempts at all-ceramic RBFDPs were initially based on a two-wing design. Numerous unilateral debonding incidents and connector fractures have been observed. Such technical complications have been explained through ceramics’ lack of plastic deformation potential (brittle material), leading to further studies on a cantilevered design for all-ceramic RBFDPs to overcome these issues. At the University of Kiel, Kern et al determined a 10-year survival for their study’s cantilever group (zirconia or alumina infiltrated ceramic) at 94.4%, compared with that of their study’s two-wing group at 67.3%. 5 These authors also stated that zirconia ceramic RBFDPs yielded a 10-year survival rate of 98.2%, without any influence from the reasons for patients’ missing teeth (trauma, agenesis). 5 The University of Geneva has also focused on all-ceramic anterior RBFDPs. Naenni et al and Sailer et al successively mentioned a 100% survival rate after a 10-year follow-up for 10 zirconia resin-bonded fixed partial dentures and also after a 6-year study of 35 glass-ceramic (Empress and Emax Ivoclar Vivadent, Schaan, Liechtenstein) RBFDPs. 20 21

Additionally, the use of glass-ceramics seems promising. 15 French practitioners Tirlet and Attal have also defended the choice of glass-ceramics, citing their better optical properties and bonding potential compared with infiltrated ceramics, such as zirconia. 30 The relative weakness of glass-ceramics’ mechanical properties compared with infiltrated ceramics has led practitioners to consider a larger connection area on the abutment teeth. Notably, however, glass-ceramics’ substantial bonding properties have significantly optimized the final mechanical resistance of all-ceramic RBFDPs. A recent *in vitro* study concluded that “lithium disilicate cantilever RBFDP had comparable fracture strength to metal-ceramic RBFDP and had a significantly higher fracture strength than the zirconia RBFDP.” 31 Further, long-term clinical studies are needed to validate this conclusion about the use of glass-ceramics.

### Reasons for a Cantilever Design

According to the Roy principle about periodontal splints first stated in 1927, the teeth bordering the edentulous area differ in their physiological mobility. These differential micromovements create stresses on RBFDPs’ retainers. To limit such constraints, designing prostheses with a single axis of mobility was considered. Obviously, with only one support tooth, such interabutment stress is not possible in cantilever fixed partial dentures. 9 28 These results were confirmed *in vitro* by the University of Hong Kong. 32 The purpose of this assessment was to compare the fatigue bond strength of three-unit versus two-unit RBFDPs after cycles of high and repeated loads on their abutment analogs, simulating the repetitive dynamic loading that prosthetic restorations experience during mastication or parafunction. Within the limitations of such an *in vitro* study, the cantilevered design showed significantly higher bond strength than both tooth analogs of the fixed-fixed framework ( Table 6 ).

**Table 6:** Bond strength versus RBFDP design

Prosthesis design	Median strength ( *n* )
2-unit (cantilever)	421
3-unit loaded tooth analog	332
3-unit unloaded tooth analog	333
Abbreviation: RBFDP, resin-bonded fixed dental prosthesis.	

The cantilever design is appropriate when occlusal constraints are low and also when the abutment tooth’s stability is controlled. Thanks to periodontal proprioceptors, patients may unconsciously influence the magnitude of occlusal loads on the abutment teeth. When patients request pontics for occlusion, they perceive a degree of mobility that encourages them to restrain the occlusal loads, thus contributing to better longevity of their prostheses.

## Conclusion

RBFDPs present an excellent clinical 5-year longevity in the anterior sector when used for the right indications and according to proper clinical procedures. Currently, no consensus has been established on the ideal material for this type of restoration. The choice of material (mainly zirconia or glass-ceramics) depends on patients’ clinical situation. Trends are shifting toward the use of all-ceramic cantilever FDPs, whose design tends to limit constraints on RBFDPs’ retainers and, thus, increases their survival time. Estimated 5-year survival rates seem comparable for various types of RBFDP, but they are slightly lower than dental implants. However, benefit/risk/cost ratios are more advantageous for the adhesive prosthesis solution. Finally, all-ceramic cantilever RBFDPs can be considered a definitive therapy; furthermore, they are an optimal solution for adolescents or young adults with potential for continuous growth.

## References

[1] Kirac D Eraydin F Avcilar T Effects of PAX9 and MSX1 gene variants to hypodontia, tooth size and the type of congenitally missing teeth Cell Mol Biol 2016 62 13 78 84 10.14715/cmb/2016.62.13.14 28040065

[2] Coelho A Macho V Andrade D Augusto A Areais C Prevalence and distribution of tooth agenesis in a pediatric population: A radiographic study Rev Gaucha Odontol 2012 60 04 505 508

[3] Marinho ACMR Manso MC Colares V de Andrade DJC Prevalence of dental trauma and associated factors in adolescents of Oporto City Rev Port Estomatol Cir Maxilofac 2013 54 03 143 149

[4] Pjetursson B E Tan W C Tan K Brägger U Zwahlen M Lang N P A systematic review of the survival and complication rates of resin-bonded bridges after an observation period of at least 5 years Clin Oral Implants Res 2008 19 02 131 141 18070120 10.1111/j.1600-0501.2007.01527.x

[5] Kern M Passia N Sasse M Yazigi C Ten-year outcome of zirconia ceramic cantilever resin-bonded fixed dental prostheses and the influence of the reasons for missing incisors J Dent 2017 65 51 55 28688950 10.1016/j.jdent.2017.07.003

[6] Pjetursson B E Valente N A Strasding M Zwahlen M Liu S Sailer I A systematic review of the survival and complication rates of zirconia-ceramic and metal-ceramic single crowns Clin Oral Implants Res 2018 29 16 199 214 30328190 10.1111/clr.13306

[7] Kern M Fifteen-year survival of anterior all-ceramic cantilever resin-bonded fixed dental prostheses J Dent 2017 56 133 135 27832968 10.1016/j.jdent.2016.11.003

[8] Shahdad S Cattell M J Cano-Ruiz J Gamble E Gambôa A Clinical evaluation of all ceramic zirconia framework resin bonded bridges Eur J Prosthodont Restor Dent 2018 26 04 203 211 30398816 10.1922/EJPRD_01810Shahdad09

[9] Botelho M G Chan A WK Leung N CH Lam W YH Long-term evaluation of cantilevered versus fixed-fixed resin-bonded fixed partial dentures for missing maxillary incisors J Dent 2016 45 45 59 66 26756882 10.1016/j.jdent.2015.12.006

[10] Klink A Hüttig F Zirconia-based anterior resin-bonded single-retainer cantilever fixed dental prostheses: a 15- to 61-month follow-up Int J Prosthodont 2016 29 03 284 286 27148991 10.11607/ijp.4220

[11] Tanoue N Longevity of resin-bonded fixed partial dental prostheses made with metal alloys Clin Oral Investig 2016 20 06 1329 1336 10.1007/s00784-015-1619-9 PMC4914526 26438343

[12] Kumbuloglu O Özcan M Clinical survival of indirect, anterior 3-unit surface-retained fibre-reinforced composite fixed dental prosthesis: up to 7.5-years follow-up J Dent 2015 43 06 656 663 25913141 10.1016/j.jdent.2015.04.006

[13] Galiatsatos A A Bergou D Clinical evaluation of anterior all-ceramic resin-bonded fixed dental prostheses Quintessence Int 2014 45 01 9 14 24392490 10.3290/j.qi.a30766

[14] Spinas E Aresu M Canargiu F Prosthetic rehabilitation interventions in adolescents with fixed bridges: a 5-year observational study Eur J Paediatr Dent 2013 14 01 59 62 23597223

[15] Sun Q Chen L Tian L Xu B Single-tooth replacement in the anterior arch by means of a cantilevered IPS e.max Press veneer-retained fixed partial denture: case series of 35 patients Int J Prosthodont 2013 26 02 181 187 23476915 10.11607/ijp.3102

[16] Aggstaller H Beuer F Edelhoff D Rammelsberg P Gernet W Long-term clinical performance of resin-bonded fixed partial dentures with retentive preparation geometry in anterior and posterior areas J Adhes Dent 2008 10 04 301 306 18792701

[17] King P A Foster L V Yates R J Newcombe R G Garrett M J Survival characteristics of 771 resin-retained bridges provided at a UK dental teaching hospital Br Dent J 2015 218 07 423 428discussion 428 25858740 10.1038/sj.bdj.2015.250

[18] Lam W YH Botelho M G McGrath C PJ Longevity of implant crowns and 2-unit cantilevered resin-bonded bridges Clin Oral Implants Res 2013 24 12 1369 1374 23025467 10.1111/clr.12034

[19] Botelho M G Ma X Cheung G JK Law R KS Tai M TC Lam W YH Long-term clinical evaluation of 211 two-unit cantilevered resin-bonded fixed partial dentures J Dent 2014 42 07 778 784 24685984 10.1016/j.jdent.2014.02.004

[20] Naenni N Michelotti G Lee W Z Sailer I Hämmerle C H Thoma D S Resin-bonded fixed dental prostheses with zirconia ceramic single retainers show high survival rates and minimal tissue changes after a mean of 10 years of service Int J Prosthodont 2020 33 05 503 512 32956431 10.11607/ijp.6737

[21] Sailer I Bonani T Brodbeck U Hämmerle C H Retrospective clinical study of single-retainer cantilever anterior and posterior glass-ceramic resin-bonded fixed dental prostheses at a mean follow-up of 6 years Int J Prosthodont 2013 26 05 443 450 23998142 10.11607/ijp.3368

[22] Younes F Raes F Berghe L V De Bruyn H. A retrospective cohort study of metal-cast resin- bonded fixed dental prostheses after at least 16 years Eur J Oral Implantology 2013 6 01 61 70 23513203

[23] Boening K W Ullmann K A retrospective study of the clinical performance of porcelain-fused-to-metal resin-bonded fixed partial dentures Int J Prosthodont 2012 25 03 265 269 22545257

[24] Garnett M J Wassell R W Jepson N J Nohl F S Survival of resin-bonded bridgework provided for post-orthodontic hypodontia patients with missing maxillary lateral incisors Br Dent J 2006 201 08 527 534 17057683 10.1038/sj.bdj.4814160

[25] Chai J Chu F CS Newsome P RH Chow T W Retrospective survival analysis of 3-unit fixed-fixed and 2-unit cantilevered fixed partial dentures J Oral Rehabil 2005 32 10 759 765 16159355 10.1111/j.1365-2842.2005.01495.x

[26] Corrente G Vergnano L Re S Cardaropoli D Abundo R Resin-bonded fixed partial dentures and splints in periodontally compromised patients: a 10-year follow-up Int J Periodontics Restorative Dent 2000 20 06 628 636 11203600

[27] van Heumen C C van Dijken J WV Tanner J Five-year survival of 3-unit fiber-reinforced composite fixed partial dentures in the anterior area Dent Mater 2009 25 06 820 827 19339043 10.1016/j.dental.2009.01.103

[28] Saker S El-Fallal A Abo-Madina M Ghazy M Ozcan M Clinical survival of anterior metal-ceramic and all-ceramic cantilever resin-bonded fixed dental prostheses over a period of 60 months Int J Prosthodont 2014 27 05 422 424 25191882 10.11607/ijp.3776

[29] Patel P M Lynch C D Sloan A J Gilmour A SM Treatment planning for replacing missing teeth in UK general dental practice: current trends J Oral Rehabil 2010 37 07 509 517 20374439 10.1111/j.1365-2842.2010.02077.x

[30] Tirlet G Attal J-P Lithium disilicate reinforced glass-ceramic cantilever bonded bridges Reasons for choice and clinical use Real Clin 2015 26 03 35 46

[31] Gresnigt M M Tirlet G Bošnjak M van der Made S Attal J P Fracture strength of lithium disilicate cantilever resin bonded fixed dental prosthesis J Mech Behav Biomed Mater 2020 103 103615 32090939 10.1016/j.jmbbm.2019.103615

[32] Wong T L Botelho M G The fatigue bond strength of fixed-fixed versus cantilever resin-bonded partial fixed dental prostheses J Prosthet Dent 2014 111 02 136 141 24189114 10.1016/j.prosdent.2013.07.004

